# A comparison of nutritional intake and daily physical activity of girls aged 8-11 years old in Makkah, Saudi Arabia according to weight status

**DOI:** 10.1186/s12889-017-4506-2

**Published:** 2017-06-21

**Authors:** Rabab Al-Kutbe, Anne Payne, Anne de Looy, Gail A. Rees

**Affiliations:** 1 School of Biomedical and Healthcare Sciences, Plymouth University, Drake Circus, Plymouth, Devon PL4 8AA UK; 2 School of Health Professions, Plymouth University, Plymouth, UK

**Keywords:** Dietary habits, Energy intake, Girls, Nutrient intake, Physical activity, Saudi Arabia

## Abstract

**Background:**

Obesity rates in Saudi Arabia are amongst the highest in the world. It is known that teenage girls are less active than teenage boys, but less is known about the diet and activity patterns in younger girls. Therefore this study sought to investigate dietary intake and daily physical activity in girls aged 8-11 years old in Saudi Arabia.

**Methods:**

This was a cross- sectional observational study conducted in seven schools across the city of Makkah. A total of 266 girls had anthropometric measurements taken including height, weight, waist circumference and body fat estimations. Dietary assessment using a 4 day unweighed diet diary was undertaken in 136 of these participants, and 134 agreed to monitor their physical activity for the 4 days using an accelerometer. After exclusion for under-reporting, 109 remained in the dietary analysis and 78 in the physical activity analyses. Differences in means between BMI groups were determined using one-way ANOVA with post hoc Tukey test. Multivariable linear regression analysis was performed to look at the effect of multiple variables on body weight.

**Results:**

A total of 30% of participants were classified obese or overweight. There was a significant difference in the mean daily energy intake between the BMI groups with the obese group having the highest energy, fat, carbohydrate and protein intake (obese group: 2677 ± 804 kcal/d; healthy weight group: 1806 ± 403 kcal/d, *p* < 0.001), but the percentage contribution of the macronutrients to energy intake remained the same across the BMI groups. There were no differences in number of steps taken per day or time spent in moderate to vigorous intensity exercise according to BMI category. Most of the girls did not meet daily physical activity guidelines (5969 to 6773 steps per day and 18.5 - 22.5 mins per day of moderate to vigorous activity). Multiple linear regression showed that energy intake positively predicted body weight (Beta = 0.279, *p* =0 .001), whereas, total energy expenditure per kg of body weight and family income had a significant negative influence on body weight (Beta = −0.661, *p* < 0.001; −0.131, *p* = 0.028 respectively).

**Conclusions:**

The results of this cross sectional analysis suggest that obesity in girls aged 8-11 years is linked to excessive energy intake from all macronutrients and the majority of girls in all weight categories are inactive. Research should be conducted to further investigate causal relationships in longitudinal studies and develop interventions to promote dietary change and activity that is culturally acceptable for girls in Saudi Arabia.

## Background

Obesity has become a widespread public health concern in most developed nations and the prevalence of obesity varies by region and according to sociodemographic variables [1]. According to a recent systematic review [2], Saudi Arabia has the second highest rate of obesity among females in the Middle Eastern countries of the Gulf Cooperation Council at 38.4%, with Qatar at 45.3%. This is higher than female obesity rates in England (27%) and the USA (35.8%). Saudi Arabia has changed very rapidly since the 1960s. The country has developed economically, and with it has come profound social and lifestyle changes [3]. For instance, economic transitions facilitate access to modern transportation, western imports as well as the availability of fast food [3]. In 2002 El-Hazmi et al. [4] found higher rates of overweight (27.6%) and obesity (13.8%) in the eastern oil-field provinces and lower rates in the mountainous south-west (11.0% and 4.3% respectively), where lifestyles are more traditional.

The prevalence of overweight and obesity in children in Saudi Arabia has been estimated at 23.1% and 11.3% respectively [5]. Tackling obesity in children is important as it might reduce the risk of adulthood obesity [3]. Research has also shown that being overweight or obese in childhood is associated with mental and psychological health problems such as depression, anxiety and low self-esteem [6], and obesity adversely affects the physical health and development of the child. Increasing adiposity has been associated with higher inflammatory markers and with lower sex hormone binding globulin (SHBG) levels between the ages of 5 and 15 years [7]. Lower SHBG levels anticipated earlier puberty. In particular, excess body fat contributes to altered levels of sex hormones, and this, in turn, seems to be a major contributor to the earlier onset of puberty, especially in girls [7]. Therefore, preventing obesity in pre-adolescents will benefit the child’s health now and in the future.

Several factors have been identified as contributing to the high incidence of obesity amongst Saudi children. As in many other countries worldwide, a general lack of physical activity, sedentary behavior from prolonged use of computers and screen-based devices, and watching television while simultaneously eating energy-dense snacks have been found to be common [3, 8]. A previous study has suggested that 60% of children in Saudi Arabia do not participate in sufficient physical activity [9]. A recent large-scale study in Saudi Arabia highlighted the problem of poor dietary habits and sedentary behaviour in teenagers aged 14-19 years [10]. By this age, children are making their own lifestyle choices and some girls will enter into marriage after the age of 16 years.

There is less information available about the dietary and exercise habits of younger children, particularly girls of differing weight status. There are cultural restrictions for girls and women that make exercise less accepted and the climate restricts outdoor activity. Excessive body mass has been shown to have many adverse consequences for all phases and aspects of reproduction such as complications for the birth and obesity negatively affects the mother’s health during pregnancy [11]. Therefore it is particularly important to prevent obesity in girls prior to the adolescent period.

Previous studies have not undertaken anthropometric measurements and prospective assessment of activity and diet simultaneously in girls of this age group. Therefore, with particular attention to robust methods of measurement, this study sought to determine how nutritional intake, and amounts of physical exercise differed according to weight status in primary-school girls aged 8-11 years old in the city of Makkah in western Saudi Arabia.

## Methods

### Study sample

This was a cross-sectional school-based study. Seven girls’ schools (3 public and 4 private) were nominated by the Makkah Education Department, different areas being selected so as to obtain a sample representative of the city’s school population. Schools gave their consent for the research to take place and information and consent sheets were given to all 2nd, 3rd, 4th and 5th grade students (aged 8-11 years old). Parent and child gave written informed consent to participate in the study and children were allowed to withdraw themselves from the study any time. Approval was granted by the Faculty of Science and Technology Research Ethics Committee at Plymouth University and The School Health Affairs Committee in Saudi Arabia. Permission was also granted by the General Directorate of School Education in Makkah.

#### Anthropometric and body-fat measurements

All measurements were conducted in the school laboratories by one researcher (January to May 2014). Using a portable stadiometer (Seca, UK), participants stood with bare feet and height was measured to the nearest 0.1 cm. Each participant’s information (height, age and gender) were entered into the Tanita Body Composition Analyser (TBF-300 M/TBF-300MA, Birmingham, UK) to calculate BMI [weight (kg) / height (m)^2^] and body-fat percentage. The BMI was plotted on the 2000 CDC growth reference curve to classify the children into obese (95 > centile), overweight (86-94 centile), normal weight (5-85 centile), and underweight (<5 centile). The CDC reference scale was considered valid for use with Saudi children as it had been validated against the WHO growth reference curves [12]. Waist circumference was measured to the nearest 0.1 cm by means of a non-stretch tape-measure positioned 4 cm above the umbilicus. All measurements were taken once by one trained nutritionist.

#### Diet diaries

Diet diaries, developed by the WAKEUP Study Group Peninsula Medical School [13], were adapted for children’s diets in Saudi Arabia and piloted on children of the study age-range. In response to pilot feedback, adjustments were made to include pictures for estimating portion sizes for some meals and drinks, and the diary was translated into Arabic. Girls were asked to complete their diaries for 4 days using standard household measurements. Participants logged their food intake over two school days and then on the 2 days of the weekend (excluding special occasions e.g. parties). Each student was interviewed daily during the week by the nutritionist to ensure that they had logged the details correctly. After the weekend they were interviewed again to review their diary entries. The diets were recorded with accompanying explanations of the quantity and then dietary data were analysed using Arabic food-composition tables’ software (Arab Food Analysis Programme, 1st version, 2007).

#### Physical activity methods

To record physical activity, accelerometers (WGT3X-BT Actigraph, Fort Walton, Florida) were worn for the 4 days that diet was recorded. These monitors have previously been assessed for their validity in another epidemiological study on children [14]. Each participant wore a belt to which the accelerometer was attached on the right site. These devices were removed only when the girl was washing or in bed; otherwise, they recorded the child’s total level of activity and number of steps taken throughout the day. The intensities of physical activity were measured according to Evenson cut points [15]. Data were excluded if there was less than 10 h per day wear time over 3 days [15, 16].

### Calculation of the total energy expenditure (TEE) via the METs

The METs rate was calculated using the Freedson et al. (2005) [17] equation obtained from the accelerometer data. The METs average was multiplied by the estimated BMR in order to estimate TEE.

#### Identification of under-reporters of dietary intake

Participants who under-reported their diet were identified by applying the formula energy intake (EI) < basal metabolic rate (BMR) ×1.2 which has been used by Schutz et al. [18] to indicate a cut-off point. BMR was estimated using the Henry equations [19]. Therefore, using the food diaries, a calculation was made for the average energy intake over 4 days, and if EI was < BMR × 1.2, the participant’s food and nutrient intake data were excluded from the analysis.

#### Data analysis

The data were coded and entered into SPSS analysis program (IBM SPSS Statistics version 20). The data are presented as means and standard deviations. Differences in means were determined using one-way ANOVA (for more than two groups with post hoc Tukey test). Pearson correlation coefficients were calculated for evaluating the relationships between the factors. Differences in age and anthropometric measures between those undertaking just the body composition measurements and those also participating in the dietary analysis were assessed using an independent T-test. A multiple linear regression was calculated to predict the body weight in the girls based on the daily energy intake, age, family income, number of steps/d and TEE per kg body weight. A level of significance of *p* ≤ 0.05 was used.

## Results

**Fig. 1:**
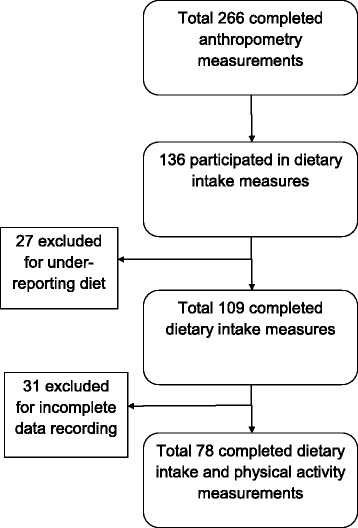
Number of subjects in each measurement

Four hundred and seven students were invited to participate, and of these 266 (65.3%) students agreed to be involved in the anthropometric measurements. A total of 109 of these participants completed the diet diary and 78 completed the physical activity components of the study (Fig. 1).

### Body composition

As shown in Table 1, there were highly statistically significant differences in all elements of body composition between BMI groups. Unsurprisingly, obese subjects had significantly greater waist circumferences, with a maximum 104 cm, and there was an overall increase in WC across the BMI groups. A total of 30% of the study population was found to be overweight or obese.

**Table 1:** Body composition and anthropometric measurements in underweight (UW), healthy-weight (HW), overweight (OW), and obese (OB) girls. *n* = 266

Body composition	UW	HW	OW	OB
	*N* = 43	*N* = 143	*N* = 34	*N* = 46
Age (years)	9.5 ± 1.27	9.6 ± 1.1	9.9 ± 1.2	9.3 ± 1.2
Body Fat %^***^	9.9 ± 4.4^a^	18.1 ± 4.6^b^	29.5 ± 3.4^c^	36.7 ± 3.9^d^
Weight (kg)^***^	22.9 ± 4.8^a^	29.3 ± 6.4^b^	41.4 ± 7.1^c^	50.3 ± 11.4^d^
WC (cm)^***^	53.7 ± 4.0^a^	59.2 ± 5.7^b^	72.1 ± 5.3^c^	79.8 ± 7.9^d^
Height (cm)^***^	130.1 ± 11.6^a^	132.9 ± 10.4^a,b,c^	138.5 ± 8.4^a,b,c^	138.7 ± 10^c^
BMI Percentile^***^	1.6 ± 0.9^a^	39.5 ± 23.8^b^	90 ± 3.6^c^	97.9 ± 0.9^d^
Height Percentile^***^	34 ± 32.1^a^	46 ± 30.7^b^	59.6 ± 27^c^	72.5 ± 26.1^d^

Data are means and standard deviations

One way ANOVA: ***significant differences *p* < 0.001 between BMI groups

^a,b,c,d^Tukey Post hoc test: means with the same letter indicate no significant difference. Any difference between two means carrying different letters is significant at 1%

There was a positive correlation between the height and waist circumference (Pearson’s *r* = 0.487, *p* < 0.001); that is, WC increased with height. Similarly, body fat percentage and height were positively correlated (Pearson’s *r* = 0.36, *p* < 0.01).

An independent T-test was carried out on the age, weight, height and waist circumference between those only undertaking the body composition measurements (*n* = 157) and those who also agreed to record their diet (*n* = 109). There were no significant differences between the two groups in age (9.6 vs 9.5 years) and height (135.6 vs 133.8 cm). However, those girls who only took part in the anthropometric measures were significantly heavier (37.4 vs 32.4 kg, *P* < 0.002) and had a larger mean waist circumference (67.9 vs 62.7 cm, *P* < 0.020) than those who also agreed to record their diet.

#### Dietary intake

The total number of subjects who completed the diet diary was 109, all with body-composition measurements completed. A total of 27 subjects were found to have EI lower than BMR ×1.2 and were excluded from the analysis (Fig. 1).

Table 2 displays the nutrient intake according to BMI categories. It also shows the contribution of different food types to energy intake, and the daily consumption of fruit and vegetables. Average energy intake and other macronutrient intake increased gradually according to weight category (Table 2).

**Table 2:** Nutrient intake, and selected food consumption in underweight (UW), healthy-weight (HW), overweight (OW), and obese (OB) participants. *n* = 109

Average for 4 Days	UW	HW	OW	OB
	*N* = 11	*N* = 50	*N* = 16	*N* = 32
Age (years)	9.5 ± 1.27	9.6 ± 1.1	9.9 ± 1.2	9.3 ± 1.2
Energy (kcal)^***^	1412 ± 291^a^ (1133-1909)	1806 ± 403^a^ (1207-2697)	2489 ± 887^b^ (1438-5168)	2677 ± 804^b^ (1595-5168)
EI (Kcal/kg Body wt)*	66 ± 20.32 (47.1 -117.16)	62 ± 16.8 (37.17 - 98)	64 ± 26.6 (37.58 -127.62)	53 ± 14 (27.7 - 89.88)
EI Kcal/kg FFM)	68 ± 10.2 (53.2-82.1)	75 ± 19.3 (45.1- 123.6)	80 ± 25.6 (54.4- 112.12)	86 ± 20.5 (53.5- 143.9)
Protein (g/d)***	52.9 ± 19^a^ (36.2-86.8)	66.3 ± 24^a^ (25.4-118.2)	79.1 ± 39^a,b^ (39.2-213.3)	87.8 ± 35^b^ (37.7-213.3)
Fat (g/d)^***^	50.5 ± 11^a^ (35.8-68.7)	68.8 ± 24.3^a^ (5.3-117.8)	104.5 ± 39^b^ (52-209.2)	108.2 ± 44^b^ (50.0-211.2)
CHO (g/d)^***^	199.2 ± 42.4^a^ (161.4-276)	236.5 ± 62.3^a^ (53.6-435.5)	347.4 ± 109.9^b^ (191.8 -642.2)	362 ± 93^b^ (233.7-642.2)
Fruits (g/d)	28.9 ± 39.5 (0.0-105)	35.2 ± 43.5 (0.0-200.5)	29.5 ± 54.8 (0.0-200)	32.9 ± 72.7 (0.0-393.7)
Vegetables (g/d)^**^	18.3 ± 22^a^ (.00-61.2)	52.3 ± 44.4^a^ (.00-220)	105 ± 114.7^b^ (.00-400)	84.3 ± 73.8^a,b^ (.0-348.7)
Sweets (g/d)^***^	59.2 ± 37.5^a^ (.0-145)	98.5 ± 53.4^a^ (.00-237.5)	129.9 ± 67.4^a,b^ (12-253)	147.7 ± 97.7^b^ (.0-361.7)
Sugary drink (ml/d)^***^	64.4 ± 40.6^a^ (.0-125)	84.6 ± 51^a^ (.0-212)	104.4 ± 70.4^a,b^ (.0-250)	124.2 ± 52.2^b^ (42-283)
Savoury Snack (g/d)^*^	1.5 ± 3.5^a^ (0.0-8.7)	20 ± 24.5^a, b^ (0.0-120)	22.7 ± 3.6^a, b^ (0.0- 125)	37 ± 44^b^ (0.0-187)

*** Significant differences *p* < 0.001; *significant differences *p* < 0.05 for the effect of the BMI groups

^a,b,c^Tukey Post hoc test: means with the same letter indicate no significant difference. Any difference between two means carrying different letters is significant at 1%

”Sweets” include: chocolate, sweets, biscuits, cakes, desserts and ice cream

”Sugary drinks” include: carbonated drinks, fruit squash, and juice with added sugar

”Savoury snacks” include: crisps and popcorn

The healthy-weight group showed the highest average fruit intake whereas the underweight group reported the lowest, although the difference was not statistically significant. With regard to vegetable consumption, the highest intake was recorded by the overweight group, and the underweight group recording the lowest. As can be seen in Table 2 there was a noticeable pattern in the consumption of sugary drinks, sweet, and savoury snacks, with the highest intake in the obese girls and the lowest in the underweight group.

The differences between the energy intake (EI) for each BMI group were calculated by comparing means using ANOVA and a post hoc (Tukey) test. There was a significant difference (*p* < 0.05) between all groups, except between the overweight and obese groups which showed no difference (*p* = 0.327). There were no significant differences between the groups for the EI per kg of FFM, although there was a gradual increment across the BMI groups, but EI per kg BW was different between the groups (66 kcal/kg BW in underweight to 53 kcal/kg BW in obese group) (Table 2).

In order to assess the energy intake from different sources, a breakdown of nutrient and selected foods are shown in Table 3. Energy provided by fat, protein and carbohydrate increased across the BMI groups, but once corrected for energy intake (% of total energy), there were no differences between groups. Likewise for savoury snacks and sugary drinks, overweight and obese groups consumed more than the healthy weight and underweight groups, but the percentage contribution to energy intake did not differ between the groups. The obese group obtained more than 6 % of their total daily energy intake from savoury snacks alone but this was not significantly different to the other groups.

**Table 3:** The contribution of macronutrients and selected foods to energy intake in underweight (UW), healthy-weight (HW), overweight (OW), and obese (OB) participants. *n* = 109

Average for 4 Days	UW	HW	OW	OB
	*N* = 11	*N* = 50	*N* = 16	*N* = 32
Energy (kcal)^***^	1412 ± 291^a^ (1133-1909)	1806 ± 403^a^ (1207-2697)	2489 ± 887^b^ (1438-5168)	2677 ± 804^b^ (1595-5168)
Energy from protein (kcal)^***^	211 ± 76^a^ (132-347)	264 ± 100^a^ (102-473)	312 ± 159^a,b^ (157-853)	359 ± 137^b^ (202-853)
% energy from protein	14.2% ±3.2 (10.6-20.8)	14.5% ±3.9 (5.4-25.8)	12.2% ±1.9 (9-16.5)	13.5% ±2.9 (7.9-17.9)
Energy from Fat (kcal)^***^	454 ± 99^a^ (618-322)	624 ± 211^a^ (200-1061)	940 ± 351^b^ (468-1883)	974 ± 396^b^ (450-1901)
% energy from fat	32.2% ±4 (28.1-41.7)	34% ±6 (12.5-42.9)	38.2% ±9.1 (27.5-67.9)	35.6% ±5.8 (27.4-47.4)
Energy from CHO (kcal)^***^	796 ± 169^a^ (646-1104)	946 ± 249^a^ (214-1742)	1389 ± 439^b^ (767-2569)	1442 ± 370^b^ (935-2563)
% energy from CHO	56.4% ±3.5 (49.5-63)	52.6% ±9.2 (13.26-67.8)	57% ±12.5 (39.4-98.6)	54.7% ±6.1 (39.6-67.9)
Energy from Savoury Snacks (kcal)^**^	8 ± 17.7^a^ (.00-44)	100 ± 123^a,b^ (.00- 600)	117 ± 167^a,b^ (.00- 625)	186 ± 220^b^ (.00-937)
% energy from Savoury Snacks	0.6% ±1.4 (.00-3.60)	5.6% ±6.8 (.00-28.8)	4.8% ±7.1 (.00-27)	6.2% ±6 (.00-23)
Energy from sugary drinks (kcal)^**^	72 ± 44^a^ (21- 121)	120 ± 94^a^ (26-326)	158 ± 112^a, b^ (42- 292)	144 ± 49^b^ (68- 221)
% energy from sugary drinks	4.5% ±3 (.00-11)	4.6% ±2.9 (.00-13.1)	4.2% ±3.1 (.00-11.3)	4.7% ±2 (2-10.60)

***Significant differences *p* < 0.001; **significant differences *p* < 0.01 for the effect of the BMI groups

^a,b,c^Tukey Post hoc test: means with the same letter indicate no significant difference. Any difference between two means carrying different letters is significant at 1%

### Number of steps and total energy expenditure (TEE)

As expected, there was a highly statistically significant increase in TEE as BMI increased, but there was no significant difference in minutes spent in moderate to vigorous intensity of physical activity between the BMI groups. The underweight group recorded the highest average minutes per day in vigorous activity (4 mins/d), whereas the overweight spent less time (1.5 mins/d). The highest percentage of time in sedentary activity was spent by the obese group, but it was not significantly different to the other groups (Table 4). There were no significant differences in the average number of steps per day between the BMI groups. The mean value for the whole group was 6757 steps/day and only 9.1% of the girls took 10,000 steps or more per day.

**Table 4:** Physical activity in underweight (UW), healthy-weight (HW), overweight (OW), and obese (OB) girls *n* = 78

	UW	HW	OW	OB
	*n* = 7	*n* = 42	*n* = 10	*n* = 19
Total energy expenditure (kcal / day)***	1501.8^a^ ± 130.7 (1390-1768)	1602.9^a^ ± 220.6 (1315-2421)	1725.4^a,b^ ± 218.9 (1448-2157)	1908.7^b^ ± 323.6 (1378-2779)
TEE/ body weight/d (kcal/kg/d)***	70.9^a^ ± 8.3	56.4^b^ ± 11.2	43.8^c^ ± 7.27	37.8^c^ ± 6.7
Minutes in sedentary PA >100 CPM	426.07 ± 138.99	442.33 ± 173.15	513.55 ± 186.37	466.44 ± 156.90
Minutes in light PA 101 - 2295 CPM*	335.64^a^ ± 95.15	366.87^b^ ± 98.36	392.57^b^ ± 115.91	302.18^a^ ± 98.74
Minutes in moderate PA 2296 - 4011 CPM	18.10 ± 11.21	17.26 ± 8.83	14.90 ± 7.91	16.22 ± 6.89
Minutes in vigorous PA 4012 - ∞ CPM	4.42 ± 6.31	3.65 ± 2.97	1.5 ± 1.2	2.26 ± 1.5
Minutes in moderate to vigorous PA <2296 CPM	22.53 ± 16.5 (4.5-53.5)	20.91 ± 11.2 (5-51.25)	18.6 ± 7.5 (6-28.50)	18.4 ± 7.7 (4.50-33.75)
Steps / day	6406 ± 2805.7 (3087-11,479)	6773 ± 1788 (3250-12,480)	6097 ± 2324.5 (2458-8726)	5969 ± 1786.6 (2322-8512)

^***^Significant difference (*p* < 0.001); *significant difference (*p* < 0.05)

^a,b,c^Tukey Post hoc test: means with the same letter indicate no significant difference. Any difference between two means carrying different letters is significant at 5%

For light activity, there was a significant difference; the results of the post-hoc test showed the differences were between the obese group and healthy and underweight groups

Table 5 shows a highly statistically significant difference in energy balance between the groups with the underweight group showing negative energy balance, a small positive energy balance in the healthy weight group, but increasingly large positive energy balance in the overweight and obese groups.

**Table 5:** Estimated energy balance in underweight (UW), healthy-weight (HW), overweight (OW), and obese (OB) girls *n* = 78

	UW	HW	OW	OB
	*n* = 7	*n* = 42	*n* = 10	*n* = 19
Energy intake (kcal/d)^***^	1299.14 ± 204.65	1724.61 ± 387.62	2239.30 ± 608.51	2741.84 ± 845.51
Energy expenditure (kcal/d)^***^	1501.8 ± 130.7 (1390.9-1768.1)	1602.9 ± 220.6 (1315.1-2421.6)	1725.4 ± 218.9 (1448.6-2157)	1908.7 ± 323.6 (1378-2779.5)
Energy storage (kcal/d)^***^	−202.73 ± 248.15	121.70 ± 445.73	513.84 ± 749.53	833.13 ± 860.52

***significantly different (*p* < 0.001)

Preliminary analyses were conducted to ensure no violation of the assumptions of normality, multicollinearity and the outliers. A multiple linear regression was calculated to predict body weight in the girls based on daily energy intake, number of steps/d, daily TEE per kg body weight, age and family income. It was found that these variables explain a significant amount of the variance in the weight (F (4, 73) = 48.07, *p* < 0.001, R2 = 0.770). The analysis showed that daily number of steps and age did not significantly predict weight (Beta = 0.034, 0.058), *p* = 0.575, 0.368 respectively). However, EI positively predicted body weight (Beta = 0.279, *p* = 0.001), whereas, TEE/BW (kg) and income had a significant negative influence on body weight (Beta = −0.661, −0.131, *p* < 0.001, *p* = 0.028 respectively).

## Discussion

To the best of the authors’ knowledge this is the first study measuring physical activity in girls objectively using accelerometers and recording diet prospectively in Saudi Arabia. An important finding of this study is the very low levels of total and moderate to vigorous intensity physical activity in girls aged 8-11 years in all of the BMI groups. None of the girls reached the recommendation of 60 min of moderate to vigorous activity per day and there were no differences between the groups in amount of moderate to vigorous activity. The number of steps per day was not a significant predictor of weight, but importantly less than 10% of all girls met the recommended daily number of steps (10000-12,000 steps/day). A study of Saudi boys aged 8-12 years reported that the mean number of daily steps per day for obese boys was 12,682 [20]; double the number of steps found in the obese girls in our study. Research in western countries [21] report that the average number of daily steps of girls aged 6-12 years was 12,000/d – again almost double that recorded here.

There are several possible explanations for this. One is that boys are more likely to play vigorous sports and games during their free time at school or at home [22]. Also, girls in Saudi Arabia are not able to play publically and are more confined at home and school. Public schools in Saudi do not have physical education programs and there are no public or private activity centres where young children can exercise. Any facilities would need to be separate for males and females.

Another consideration is that Makkah is regarded as a holy city, a place where citizens (including children) are expected to behave with decorum which negatively affects physical activity level. The climate is another consideration as it is too hot for long walks or more vigorous outdoor activity.

The implications of this study are that the inability of girls to exercise lead to increased risk of obesity and to the many forms of adult ill-health and morbidity that can follow. Several studies have observed a positive relationship between physical activity, physical fitness, and academic performance [23].

Another important finding from this study was the clear differences in dietary intake between the BMI groups, with obese girls consuming more total energy, sweet snacks and sugary drinks than healthy weight girls. It was estimated that overweight and obese girls were in positive energy balance by over 500 and 800 kcal/d respectively. The average energy intake recorded by obese and overweight girls was 25% higher than the UK energy recommendations for this age group [24].

Total intakes of fat, protein and carbohydrate increased significantly over the BMI groups but the percentage contribution to energy remained the same. The percentage contributions of macronutrients to energy are broadly in line with recommendations (> 50% of energy from carbohydrate; 35% energy from fat (UK, DRVs [24]). However, the UK Department of Health [24] and WHO recommended that energy intake from free sugars should not exceed 5 % of the total daily energy, but in this study the average energy intake from sugary drinks alone was 4-5% in all groups.

High consumption of sugary drinks has been observed in studies of older girls in Saudi Arabia and this has been correlated to increases in waist circumference [25]. Al-Hazzaa et al. (2011) [10] found one third of girls aged between 14 and 19 years consumed sugary drinks daily and 60% consumed them 3 to 6 times per week. Similar findings were reported by Shaath et al. [26] who found that 74.5% of girls aged 12–18 years drank sweetened carbonated beverages daily. Our research shows that sugar sweetened beverage consumption is too high in younger girls as well and contributes substantially to energy intakes. Strategies need to be put in place to reduce consumption at school, to work with parents on restricting the consumption at home and to more widely promote healthier choices.

The Ministry of Health in Saudi Arabia recommend 6 servings of fruit and vegetables per day for this age group, however the weight of a recommended portion is not specified [27]. For most girls the daily consumption of fruit and vegetables was markedly less than the daily portions recommended for UK children in the UK i.e. between 200 and 400 g/d [24]. Teenage girls in Saudi are also known to have low intakes [10] with 15% not eating any on a daily basis [28].

The finding that income was negatively associated with weight in the regression analysis was not in agreement with other studies conducted in Saudi Arabia. Other studies have all found that high SES correlated positively with obesity levels [29–31]. However this is similar to what have been found in the UK and USA [32]. There are different measures of socio-economic status and these require further investigation if comprehensive interventions are to be developed and targeted at those most in need.

The prevalence of overweight and obesity found in this study (30%) is in agreement with the findings of a study conducted in Riyadh city in Saudi Arabia in 2015 where 31.7% of girls aged 8-15 years old were overweight and obese (16.8%, 14.9% respectively) [33]. Also the average waist circumferences for overweight (72 cm) and obese girls (79 cm) are very similar to a study conducted Riyadh where overweight and obese girls (10-11 years) had an average waist measurement of 70 cm, and 78 cm respectively [25]. Therefore, although our sample was relatively small and from one city, it can be considered representative of the expected range of body compositions of girls of this age in Saudi Arabia.

### Limitations to this research

A disadvantage of the dietary-analysis programme used in this study was that it has no capability to distinguish between starch and sugars, or between non-milk extrinsic sugars (NMES) and intrinsic sugars, and so an overall estimation of NMES could not be made. However, it included many of the Arabic foods required for this research.

Many children who were invited to take part declined or did not record their diet and activity which reduced the sample size and representation of the population. Those girls who only took part in the anthropometric measures were significantly heavier and had a larger mean waist circumference than those who also agreed to record their diet. Therefore some selection bias was introduced, which is difficult to overcome. There was some under-reporting of dietary intake but obese and overweight children did not under-report their diet any more than healthy weight girls. The wear time of the accelerometers might also have been insufficient for some individuals and therefore under-estimated some of the physical activity, although those obviously under-reporting were removed from the analysis. Also, as this was a cross sectional analysis, inference about causation of obesity should be made with caution.

## Conclusion

The findings of this study have a number of important implications for prevention of childhood obesity in Saudi Arabia. Findings suggest that obesity in girls of this age is linked to excessive energy intake from all macronutrients. Sugary drinks and snack foods were consumed in high quantities and contribute to positive energy balance. Although there was no relationship found between weight and the number of steps or the time spent in moderate to vigorous activity, physical activity was exceptionally low in all weight categories.

This work highlights the need for government policy to address the issues of inadequate activity and poor diet in girls of this age. Priority needs to be given to the provision of facilities and sports teachers to enable young girls to participate in games and sports. Attention needs to be given to providing healthy school meals and promotion of a healthier diet to parents and children. Further investigations are required to formulate and evaluate interventions targeted to address excess energy intake, low fruit and vegetable consumption and the low physical activity in girls in Saudi Arabia.

## Abbreviations

BMI: Body Mass Index
BMR: Basal Metabolic Rate
CDC: Centres for Disease Control
DH: Department of Health (UK)
DRV: Dietary References Values
EI: Energy Intake
NMES: Non-Milk Extrinsic Sugars
TEE: Total Energy Expenditure
WHO: World Health Organisation.
